# Integrating Regular Exergaming Sessions in the ExerCube into a School Setting Increases Physical Fitness in Elementary School Children: A Randomized Controlled Trial

**DOI:** 10.3390/jcm11061570

**Published:** 2022-03-12

**Authors:** Sascha Ketelhut, Lisa Röglin, Anna Lisa Martin-Niedecken, Claudio R. Nigg, Kerstin Ketelhut

**Affiliations:** 1 Institute of Sport Science, University of Bern, 3012 Bern, Switzerland; claudio.nigg@unibe.ch; 2 Institute of Sport Science, Martin Luther University Halle-Wittenberg, 06120 Halle (Saale), Germany; li.roeglin@gmail.com; 3 Institute for Design Research, Department of Design, Zurich University of the Arts, 8005 Zurich, Switzerland; anna.martin@zhdk.ch; 4 Faculty of Natural Science, MSB Medical School Berlin, 14197 Berlin, Germany; kerstin.ketelhut@medicalschool-berlin.de

**Keywords:** exergaming, school-based intervention, physical fitness, health-related fitness, children

## Abstract

This study aimed to investigate the effects of a school-based exergame intervention on anthropometric parameters and physical fitness. Fifty-eight students (10.4 ± 0.8 years; 48% girls) were randomized into an intervention (IG) and a control (CG) group. Both groups participated in regular physical education classes during the three-month intervention period. The IG additionally received a 20-minute exergame intervention twice per week. At baseline and following the intervention period, body mass index (BMI) and waist-to-height ratio (WHtR) were assessed. Furthermore, a sprint test (ST), a countermovement jump test (CMJ), and a shuttle run test (SRT) were performed. Due to prescribed quarantine measures, only 34 students (18 IG; 16 CG) were included in the final analysis. A significant group–time interaction was determined in CMJ performance (*p* < 0.001; η^2^ = 0.403), with a significant increase (+2.6 ± 2.4 cm; *p* < 0.001; η^2^ = 0.315) in the IG and a significant decrease (−2.0 ± 3.1 cm; *p* = 0.009; η^2^ = 0.190) in the CG. Furthermore, ST performance significantly improved in the IG (−0.03 ± 0.08 s; *p* = 0.012; η^2^ = 0.180) but not in the CG (0.13 ± 0.16 s; *p* = 0.460; η^2^ = 0.017), revealing significant interaction effects (*p* = 0.02; η^2^ = 0.157). Significant group–time interaction was observed for the SRT (*p* = 0.046; η^2^ = 0.122), with a significant increase (+87.8 ± 98.9 m; *p* = 0.028; η^2^ = 0.147) in the IG and no changes (−29.4 ± 219.7 m; p = 0.485; η^2^ = 0.016) in the CG. Concerning BMI (*p* = 0.157; η^2^ = 0.063) and WHtR (*p* = 0.063; η^2^ = 0.114), no significant interaction effects were detected. School-based exergaming is a suitable tool to influence students’ physical fitness positively.

## 1. Introduction

Activity guidelines recommend that children engage in at least 60 minutes of moderate- to vigorous-intensity physical activity (PA) per day to improve cardiovascular and metabolic health [1]. Despite the well-established positive effects of regular PA, 41% of children and adolescents worldwide fail to meet the current activity guidelines [2], placing them at risk for future non-communicable chronic diseases [3,4,5]. Thus, strategies to promote regular PA in children are highly warranted. 

Physical fitness is considered a key determinant for promoting PA [6], as it is a foundation for competently performing PA [7]. Without a certain physical fitness level and basic competencies in different motor skills, children are limited in the amount and range of PA they can undertake [8]. According to the literature, there is a strong association between motor skill performance, physical fitness, and PA participation [9,10]. Therefore, strategies aiming to improve children’s PA need to ensure children have the competency to be physically active in the first place [11]. Especially in western industrialized countries, physical fitness levels in children are a growing concern among health professionals and physical educators [11,12]. Even though physical education (PE) classes are designed to offer opportunities to improve physical fitness, children often do not develop proficient fitness skills through PE alone [11,13]. The lack of effectiveness seen from standard PE classes demands effective intervention approaches that provide further opportunities to improve children’s physical fitness. 

An innovative and motivational approach to promoting PA and developing physical fitness in a younger demographic could be through exergames. Exergames are active video games that require bodily movements to play the game. Already a decade ago, health professionals and scholars proposed exergames as a promising approach to facilitate PA [14]. Due to attractive and immersive design, exergames may reach children who are not as keen to engage in more traditional forms of PA. According to the growing body of empirical research, exergames can increase energy expenditure compared to sedentary behaviors [14,15,16], as well as promote light to moderate PA [15,16,17,18,19]. Especially in overweight and obese youth, exergames have the potential to increase PA participation, reduce sedentary behaviors, and reduce body fat [20]. 

In recent years, exergames have been increasingly applied within school settings, contributing to higher levels of PA [18,21,22]. However, results of the effects on physical fitness are inconsistent [16,18,23]. Barnett et al. [23] could not show any effects of a 6-week exergaming intervention conducted after school hours on movement skills. Gao et al. [18] reported positive effects of an 8-week exergaming intervention on promoting moderate to vigorous PA; however, they could not show significant intervention effects on motor skill competence. Only Ye et al. [16] were able to show positive effects from a 9-month exergaming intervention on body mass index and muscle strength, but not on aerobic fitness. These inconsistent results may be due to differences in study designs, as well as the variety of exergames applied. Most of the exergames used in previous studies were commercially available and prioritized an appealing game design rather than the integration of functional training programs. The exercise intensity achieved in most current exergames is, therefore, too low to result in meaningful fitness level advancement [24,25].

In the present study, we applied a new, functional fitness game called the ExerCube, facilitating an adaptive, individually tailored, whole-body gaming experience that may overcome many of the weaknesses seen in previous exergames [26]. By adjusting the game’s challenge level according to the player’s individual cognitive and physical abilities, this game guarantees an individually tailored training stimulus [27]. This study aimed to determine the effects of this innovative exergame on physical fitness during a 12-week school-based intervention.

## 2. Materials and Methods

### 2.1. Study Design and Participants

The study was designed as a two-armed randomized controlled trial with an intervention period of 12 weeks. An a priori power analysis was conducted utilizing G*Power (Version 3.1.2; Heinrich Heine Universität, Dusseldorf, Germany). Assuming an effect size of 0.8 with an alpha level of 0.05, 26 participants were required for the study to have sufficient power. The study sample was recruited in August 2020 from an elementary school located in a socially disadvantaged area of Berlin, Germany. Prior to the commencement of the study, participating children and their parents or legal guardians were informed about the purpose of the study, and written guardian consent was obtained. Children were eligible to participate if they (1) provided written informed parental or guardian consent, (2) had no physical limitations to exercise, and (3) were currently in the fifth or sixth grade. A cohort of 58 students was included in the study. The study was reviewed and approved by the Research Ethics Board of the Medical Center Berlin (2020-09-RK1).

After a baseline examination, the children were randomized into an intervention (IG) and a control group (CG). The principal investigator carried out the randomization using a computer-generated random number table. During the three-month intervention period, the IG and the CG participated in their normal PE classes twice a week. In addition to the standard PE classes, the IG participated in two additional exergaming sessions per week. 

### 2.2. Baseline Assessment

Before and after the intervention, body mass index (BMI) and waist-to-height ratio (WHtR) were determined. Additionally, a 20-meter sprint test (ST), a countermovement jump test (CMJ), and a shuttle run test (SRT) were performed.

Pre- and post-tests were conducted in the morning hours at the school gym. Before the test procedure, the children participated in a 5-min guided warm-up. The same examiners were present during pre- and post-assessments.

#### 2.2.1. Anthropometrics

The standing heights of the children were measured barefoot to the nearest 0.5 cm using a wall-mounted scale. Body mass was determined using a digital scale (BC-545N, Tanita Europe B.V., Amsterdam, The Netherlands) while the students wore light sportswear. Waist circumference was assessed to the nearest 0.5 cm at the umbilical line while the students were standing. BMI and WHtR were calculated for each student from their respective height, weight, and waist circumference measurements.

#### 2.2.2. Physical Fitness

CMJ height is a valid and reliable method widely used to measure leg power and explosiveness [28], which are essential components of children’s musculoskeletal fitness and motor skill development [29]. 

After a familiarization test, the children conducted two bilateral countermovement jumps using an Optojump photocell system (Microgate, Bolzano, Italy), a validated tool for assessing jump height [30]. Children started from an upright position with their hands on their hips. They were instructed to dip down from a standing position and then jump as high as possible during the subsequent concentric phase. The flight time was measured, and the jump height was calculated. Both jumps were performed without shoes. The better result of the two CMJ trials was used for analysis. 

Sprint tests are commonly used in school settings and have been included in several fitness test batteries to determine power and strength [31]. Sprint performance is considered an important component of physical fitness and has been shown to be a distinguishing characteristic of successful sports performance in children [32]. The 20-meter sprint times were assessed using portable electronic timing gates (KS-sport, Siebnen, Switzerland). The children were instructed to perform two 20-meter sprints separated by three minutes of rest. All students began with their front foot positioned 0.5 m behind the start line and were instructed to perform both sprints with a maximal effort. The better result of the two sprints was used for analysis.

#### 2.2.3. Aerobic Fitness

Aerobic fitness was assessed by means of a multistage 20 m shuttle run test. The SRT is respected as a reliable method to predict aerobic fitness [33]. The students were instructed to run back and forth between two lines set 20 m apart. An audio signal emitted at specific frequencies was used to set the pace. Additionally, a study staff member participated in the test, giving the children an additional reference for the pace. The initial speed was set at 8.5 km/h and increased by 0.5 km/h every minute. The students were instructed to complete as many shuttles as possible. When a student gave up due to fatigue or two consecutive failures to finish a shuttle in the allotted time, the test was concluded. The numbers of completed shuttles and stages were recorded for each student to predict aerobic fitness.

### 2.3. Exergaming Intervention 

The children in the IG participated in two exergaming sessions per week lasting 15–20 min. The sessions were integrated into the daily school schedule and took place before, between, or after classes, as well as during breaks. A study staff member coordinated and supervised all exergaming sessions. 

The ExerCube is a physically immersive exergame setting shaped like an open cube [34]. The three cushioned walls of the cube serve as a projection screen for the game scenario and a haptic interface. During the game (Sphery Racer), the player navigates an avatar along a virtual racing track by performing a variety of whole-body movement tasks. A motion-capturing system using HTC Vive Trackers attached to the wrists and ankles detects the player’s movement in three dimensions through infrared sensor technology. By analyzing the timing and accuracy of movements throughout the game, the motion capturing system guarantees a correct execution of the different movement tasks. Before each game, the system was calibrated to match the targets to the body height of the player. 

The game Sphery Racer implements six game levels, which guide the player through the workout while also gradually increasing duration. For a 15-minute session, the duration of the levels is 1:30, 2:00, 2:40, 3:50, and 5:10 min. For a 20-minute session, the duration of the levels is 1:50, 2:30, 3:20, 5:10, and 7:10 min. The levels are interspersed with short resting phases of about 30 s. The game continuously adjusts game difficulty and complexity to the player’s fitness and cognitive skills. When the player makes too many mistakes or reaches a predetermined heart rate (HR), the game’s speed slows down. HR is tracked by a Polar HR monitor (Polar Electro OY, Kempele, Finland) and a chest strap. The HR threshold was set to 90% of the individual maximum HR calculated using the formula (HRmax = 208 − 0.7 × age) from Tanaka et al. [35].

The COVID-19 measures did not affect the exergaming intervention, and the children were allowed to continue the intervention throughout the whole assessment period. 

### 2.4. Physical Education Classes

Throughout the intervention period, both the IG and CG took part in regular PE classes of 1 × 45 and 1 × 90 min per week. A certified PE teacher conducted the classes following the regular school curriculum. Due to COVID-19 measures, PE classes were prohibited in the last week of the intervention period. 

### 2.5. Statistics

Statistical analyses were performed using IBM SPSS Statistics 27.0 (SPSS Inc., Chicago, IL, USA). Differences in subject characteristics between the groups were determined using an independent samples t-test. A Levené test was used to check the homogeneity of variance. A series of two-way (groups: exergaming vs. control) ANOVAs with repeated measures (time: baseline vs. post-intervention) were performed to determine if significant differences occurred in the study outcomes. Post hoc analyses with Bonferroni’s correction were also performed. The effect size was measured by partial eta squared (η^2^). In the study, small, medium, and large effect sizes were designated as 0.01 ≤ 0.06, 0.06 < 0.14, and ≥0.14, respectively [36]. 

Test-retest reliability for both the CMJ and ST was assessed by the intraclass correlation coefficient (ICC). The criteria, suggested by Portney and Watkins [37], to evaluate reliability was calculated as: ≥0.75 (good), 0.50–0.74 (moderate), 0.26–0.50 (fair), and ≤0.25 (poor).

## 3. Results

The session attendance rates for the exergaming sessions were 94%. The attendance rates for the PE classes were 87% in the IG and 89% in the CG. No adverse events occurred during the intervention period in any of the participants. Due to two suspected COVID-19 cases and prescribed quarantine measures, three classes could not participate in the post-examination. Thus, only 34 students (18 IG, 16 CG) were included in the final analysis. Students’ characteristics are summarized in Table 1. According to age and sex-specific percentiles, eight students were classified as overweight and eight as obese. Based on WHtR, eight children showed values in the overweight range. There were no significant differences between the groups at baseline (Table 1). 

A significant group–time interaction with a large effect was determined in CMJ performance, with a significant increase (+2.6 ± 2.4 cm; *p* < 0.001; η^2^ = 0.315) in jumping height in the IG and a significant decrease (−2.0 ± 3.1 cm; *p* = 0.009; η^2^ = 0.194) in the CG. Post hoc test results revealed no significant differences between groups before (*p* = 0.285; η^2^ = 0.0.036) or after (*p* = 0.103; η^2^ = 0.081) intervention. Furthermore, ST performance significantly improved in the IG (−0.03 ± 0.08 sec; *p* = 0.012; η^2^ = 0.180) but not in the CG (0.13 ± 0.16 s; *p* = 0.460; η^2^ = 0.0.017), revealing significant interaction effects and a large effect size. Again, there were no differences between the IG and CG in ST performance before (*p* = 0.635; η^2^ = 0.0.007) or after (*p* = 0.501; η^2^ = 0.014) intervention. Significant group–time interactions and a large effect size were observed for the SRT, with a significant increase in distance covered (+87.8 ± 98.9 m; *p* = 0.028; η^2^ = 0.147) in the IG. The CG showed no changes (−29.4 ± 219.7 m; *p* = 0.485; η^2^ = 0.016) over time (Table 2). Post hoc tests revealed no differences in SRT performance before (*p* = 0.645; η^2^ = 0.013) or after (*p* = 0.311; η^2^ = 0.033) the intervention period. No significant interactions were detected for the BMI and the WHtR measurements. During the exergaming sessions, the students reached a mean HR of 175.3 ± 2.1 bpm, corresponding to 87.4 ± 0.2% of their maximal HR. The ICC revealed a good test-retest reliability for both the CMJ (0.85) and ST (0.93). 

## 4. Discussion

The present study investigated if a regular school-based exergaming intervention induced beneficial effects on physical fitness and anthropometric parameters. We presented evidence that a 12-week school-based exergaming program improved CMJ, ST, and SRT performance when compared to the CG. These findings are relevant given that physical fitness is considered a critical determinant for promoting PA [12].

The positive effects on sprint and jump performances are congruent with previous research by Smits-Engelsman et al. [38], which showed positive effects after just 5 weeks of exergaming in 6–10-year-old children. Dickinson and Place [39] reported significant improvements in standing long jump and sprint performances in children with autism (5–15 years) after a one-year exergaming intervention. Furthermore, McGann et al. [40] reported improvements in different locomotor skills (running, hopping, skipping, jumping, and sliding) after an 8-week exergame intervention in 5–6-year-old children. Other exergaming studies have failed to detect significant changes in motor skill performance in children [18,41]. A recent systematic review by Liu et al. [42] supported these inconsistent results. 

Interestingly, children in the CG showed reduced performances in CMJ after the intervention period. It is possible that governmental restrictions due to the COVID-19 pandemic (closure of sports clubs after the first six weeks of the intervention period) may have limited children’s abilities to engage in sufficient levels of PA outside of school, potentially causing this performance decline [43]. Furthermore, PE classes were prohibited in the last week of the intervention period. 

It has previously been reported that different school-based exercise interventions are able to improve aerobic fitness [44,45]. Whether exergame-based interventions can improve aerobic fitness to a similar extent remains questionable, as previous studies have claimed that the required exercise intensity during these games is insufficient to trigger relevant physiological adaptations [24,25,46]. Our study was able to show positive effects from exergaming on SRT performance, echoing some previous research [39,47]. In contrast, Ferguson et al. [48] detected non-significant changes in SRT performance after 9 weeks of exergaming in children with developmental coordination disorder. Ye et al. [21] even assessed lower aerobic fitness following an 8-week exergaming intervention. 

No significant time–group interactions were detected in BMI and WHtR. In a previous study, Staiano et al. [49] showed that 24 weeks of home-based exergaming was able to decrease BMI in obese children. Maddison et al. [50] reported a reduction in body fat percentage for children from the intervention group following a 24-week exergaming intervention. In contrast, Graves et al. [51] revealed no significant differences in body fat percentage between children participating in a 12-week exergaming intervention and a control group, while a systematic review by Norris et al. [52] reported conflicting results.

The inconsistent results regarding the respective outcomes may be associated with considerable differences in study design, enrolled participants, and the respective exergames applied. We would argue that the positive effects in this study are attributed to the innovative game setup of the exergame employed. According to a previous study in adults, the ExerCube provided a form of vigorous PA that exceeded the exercise intensity of most commercial exergames [27]. It has been widely acknowledged that exercise of higher intensity leads to greater adaptations and more pronounced health benefits [53,54], even in children [44]. The relatively high exercise intensity reached in this exergame may be explained by the fact that the game triggers a whole-body functional exercise engaging large muscle groups. Most exergames predominantly require arm movements, engaging less muscle mass. Previous studies have shown that exergames requiring lower and upper body movements reach higher exercise intensities and are the most effective in achieving meaningful levels of PA [17]. The specific execution of lower body movements (e.g., jumping, skipping, and lunging) in the ExerCube may explain the CMJ and ST performance improvements seen in this study.

Furthermore, the ExerCube uses a motion capturing system that tracks upper and lower extremities. Thus, bodily movements can be traced more precisely than most consoles that use hand-held sensors. Tracking bodily movements utilizing hand-held devices was shown to result in poor quality movement outputs [23], as well as cheating, thus hampering improvements in motor skill performance [55].

Additionally, the sheer dimension of the game setup (~9 m^2^) requires a lot of movement to reach the targets and perform the required tasks. Finally, the current exergame’s ability to track HR throughout the game and adjust the game’s speed according to a predetermined threshold may account for the higher exercise intensity seen. Based on previous results, the ExerCube is able to offer an individually tailored exercise program that guarantees a progressive training stimulus and, therefore, can promote long-term performance developments [27].

Another strength of the study may be the fact that the intervention was integrated into a school setting with sessions included in the school timetable. This could explain the good adherence (94%) retained. Previous studies have reported lower adherence and higher dropout rates with home-based [56] compared to school-based exergaming for children [57,58].

Based on the intervention duration, this study lies within the median. Shorter interventions reported significant effects on motor skills and physical fitness [38]. However, it seems that positive effects on BMI may only be attained if longer intervention periods are applied [49,50].

Our results are of relevance as inadequate physical fitness in children may result in developmental delays, possibly decreasing PA engagement [59]. Children with high levels of physical fitness report higher levels of PA, are more fit [60], and accumulate lower levels of sedentary behavior [61]. Thus, developing adequate physical fitness levels during childhood may be an important step toward establishing lifelong PA engagement [62]. Furthermore, evidence strongly associates higher physical fitness with reduced cardiovascular risk in children [63].

Even though other exercise interventions are able to improve physical fitness in children, exergaming may present an innovative and more engaging approach. Exergames take advantage of children’s fascination with computer and video interaction [64]. Moreover, the immersive and engaging experience can help distract them from physiological cues during the exercise and, thus, enhance enjoyment [65]. Therefore, exergaming is often perceived as a form of entertainment rather than exercise [66]. Since research implies that PA enjoyment is linked to higher levels of participation in PA [67], exergames may present a valuable tool to promote lifelong PA.

A further advantage of exergames, such as the ExerCube, is that they allow children of different skill levels to engage in the game. Since the ExerCube adjusts the game speed to the player’s physical and cognitive performance, it guarantees an optimal balance between both the game’s challenge and the player’s skills and between the exercise intensity and their fitness level [68]. This allows players of all skill levels to experience success. In regular sports or PE classes, inadequate motor skills and physical fitness may impede successful participation. Furthermore, children with motor deficits may feel embarrassed by their performance in normal sports or PE classes and, subsequently, withdraw from PA [69]. Well-designed exergames can help all children experience positive feedback from enjoyment and successful achievement. This is relevant as perceived physical competence is correlated with enjoyment in PA and PE [70], supports prolonged engagement, and may even increase children’s interest in new exercise experiences [71]. 

An interesting feature of exergames is that they adhere to social distancing measures, which may be particularly useful in the current COVID-19 pandemic. Exergaming interventions can be continued when group exercises or PE classes are prohibited.

### Limitations

Different limitations ought to be discussed when interpreting these results. First, the sample of this study was significantly diminished due to COVID-19 quarantine measures, meaning further large-scale studies are warranted. 

Second, the results are limited to the specific game setting (ExerCube) and the applied game (Sphery Racer). Other exergames will likely produce different effects, probably linked to the attained exercise intensity. Third, the intervention duration was relatively short. Further long-term studies would be helpful to guide future recommendations. Fourth, different COVID-19 restrictions could have affected the outside school PA engagement of the children. Finally, we only assessed the effects in elementary school children. It is not clear if older children would benefit from the intervention to a similar extent. 

## 5. Conclusions

Our findings indicated that integrating regular exergaming sessions into a school setting increased physical fitness in elementary school children. The fact that physical fitness is strongly associated with health benefits [9] and PA participation [11] underpins the significance of the present results. Furthermore, the findings supported the implementation of exergames as innovative PA programs in school settings, helping children develop a healthy and active lifestyle. This may be particularly relevant during the COVID-19 pandemic when other PA offerings, such as PE classes, are restricted.

## Figures and Tables

**Table 1:** Student’s characteristics at baseline.

Items	Total (*n* = 34)	IG (*n* = 18)	CG (*n* = 16)	
	M ± SD	M ± SD	M ± SD	*p*-Value
Boys/Girls (*n*)	17/17	8/10	9/7	
Age (yrs)	10.5 ± 0.7	10.5 ± 0.7	10.5 ± 0.6	0.894
Height (cm)	147.3 ± 7.6	147.9 ± 8.7	146.5 ± 6.3	0.591
Body mass (kg)	45.3 ± 12.4	48.2 ± 12.4	42.1 ± 11.9	0.154
Body Mass Index (kg·m^−2^)	20.6 ± 4.2	21.7 ± 4.0	19.3 ± 4.1	0.094
Waist-to-height ratio	0.46 ± 0.06	0.47 ± 0.05	0.44 ± 0.07	0.285

Values are means (M) ± standard deviations (SD). *p*-values indicate differences between the IG and CG.

**Table 2:** Changes in outcomes from before to after intervention for the intervention group (IG) and control group (CG).

Outcome	IG (*n* = 18)		CG (*n* = 16)			
	Pre	Post	Pre	Post	*p*-Values	η^2^
BMI (kg·m^−2^)	21.7 ± 4.0	21.6 ± 4.2	19.3 ± 4.1	19.7 ± 4.1	n.s.	0.063
WHtR	0.47 ± 0.05	0.46 ± 0.05	0.44 ± 0.07	0.45 ± 0.07	n.s.	0.114
CMJ (cm)	18.6 ± 5.4	21.1 ± 5.2 ***	20.5 ± 5.2	18.6 ± 3.6 **	<0.001	0.403
ST (s)	4.12 ± 0.45	4.08 ± 0.47	4.06 ± 0.35	4.18 ± 0.32	0.020	0.157
SRT (m)	450.0 ± 228.0	537.8 ± 210.5 *	498.7 ± 208.3	469.3 ± 162.3	0.046	0.122

Abbreviations: Pre—before intervention; post—after intervention; BMI—body mass index; WHtR—waist-to-height ratio; CMJ—countermovement jump; ST—sprint test; SRT—shuttle run test; η^2^ –partial eta squared. * *p* < 0.05, ** *p* < 0.01, and *** *p* < 0.001 represent changes from before to after intervention for the IG and CG. *p*-values represent interaction effects.

## Data Availability

The data presented in this study are available on request from the corresponding author.
